# Immunomodulation of human monocytes following exposure to *Lutzomyia intermedia *saliva

**DOI:** 10.1186/1471-2172-9-12

**Published:** 2008-04-10

**Authors:** Maria José Menezes, Dirceu J Costa, Jorge Clarêncio, José Carlos Miranda, Aldina Barral, Manoel Barral-Netto, Cláudia Brodskyn, Camila I de Oliveira

**Affiliations:** 1 Centro de Pesquisas Gonçalo Moniz-FIOCRUZ, Salvador, Brazil; 2 Universidade Federal da Bahia, Salvador, Brazil; 3 Instituto de Investigação em Imunologia, Salvador, Brazil

## Abstract

**Background:**

Sand fly saliva contains potent and complex pharmacologic molecules that are able to modulate the host's hemostatic, inflammatory, and immune systems. In this study, we evaluated the effects of salivary gland sonicate (SGS) of *Lutzomyia intermedia*, the natural vector of *Leishmania braziliensis*, on monocytes obtained from the peripheral blood mononuclear cells (PBMC) of healthy volunteers. We investigated the effects of sand fly saliva on cytokine production and surface molecule expression of LPS-stimulated human monocytes uninfected or infected with *L. braziliensis*.

**Results:**

Pre-treatment of non-infected human monocytes with *L. intermedia *SGS followed by LPS-stimulation led to a significant decrease in IL-10 production accompanied by a significant increase in CD86, CD80, and HLA-DR expression. Pre-treatment with SGS followed by LPS stimulation and *L. braziliensis *infection led to a significant increase in TNF-α, IL-6, and IL-8 production without significant alterations in co-stimulatory molecule expression. However, pre-treatment with *L. intermedia *SGS did not result in significant changes in the infection rate of human monocytes.

**Conclusion:**

Our data indicate that *L. intermedia *saliva is able to modulate monocyte response, and, although this modulation is dissociated from enhanced infection with *L. braziliensis*, it may be associated with successful parasitism.

## Background

Leishmaniasis is a protozoan parasitic infection transmitted by sand flies. Different species of *Leishmania *are associated with distinct clinical forms of disease. Cutaneous leishmaniasis (CL) caused by *Leishmania major *is usually benign; infection of human hosts leads to the development of a localized cutaneous lesion that eventually heals, leading to the generation of life long-immunity. In contrast, CL caused by *L. braziliensis *is distinguished from other leishmaniasis by its chronicity, latency, and tendency to metastasize in the human host [1]. In this disease, a single ulcer with elevated borders and a necrotic centre is frequently observed, and a chronic inflammatory response develops despite the paucity of parasites. In 1–5% of patients, muco-cutaneous leishmaniasis may occur due to the intrinsic ability of *L. braziliensis *to persist within lesion scars after spontaneous or chemotherapy-mediated healing and its ability to metastasize to the nasal mucosa [2-4].

*Leishmania *parasites are transmitted to the vertebrate host when the sand fly probes for a blood meal. During blood feeding, sand fly saliva, which contains a great variety of hemostatic, inflammatory, and immunomodulatory molecules (rev. in [5]), is injected into the host's skin. Sand fly saliva is also known to facilitate parasite survival [6-9]. The enhancing effects of sand fly saliva on disease development are associated with its ability to inhibit several functions of antigen presenting cells (APCs) such as antigen presentation and nitric oxide (NO) production [10-12]. We have recently shown, using an experimental model of infection, that pre-exposure to *Lutzomyia intermedia *saliva is able to enhance infection with *Leishmania braziliensis *[13]. Importantly, individuals with active cutaneous leishmaniasis also showed higher humoral immune responses to *L. intermedia *saliva compared to control subjects, suggesting an association between positive anti-saliva immune response and the development of disease. Therefore, we were interested in determining the effects of *L. intermedia *saliva in a more restricted experimental system, employing LPS-stimulated human monocytes alone or infected with *Leishmania braziliensis*. This approach has already been undertaken with *P. papatasi *[14] and with *L. longipalpis *[15], with which it was shown that saliva impairs cytokine production and co-stimulatory molecule expression on human monocytes and dendritic cells. Monocytes and dendritic cells are present in the hemorrhagic pool created by sand fly feeding and are, therefore, exposed to sand fly saliva. Because of this, we have examined the effects of saliva from *L. intermedia*, the main vector of *L. braziliensis *in Brazil, on human monocytes stimulated with LPS and uninfected or infected with *L. brazilienis*. Understanding how salivary products interfere with LPS-induced co-stimulatory molecule expression, cytokine production, and parasite infection may help clarify the complex vector-parasite-human host interplay.

## Results and Discussion

**Figure 1:**
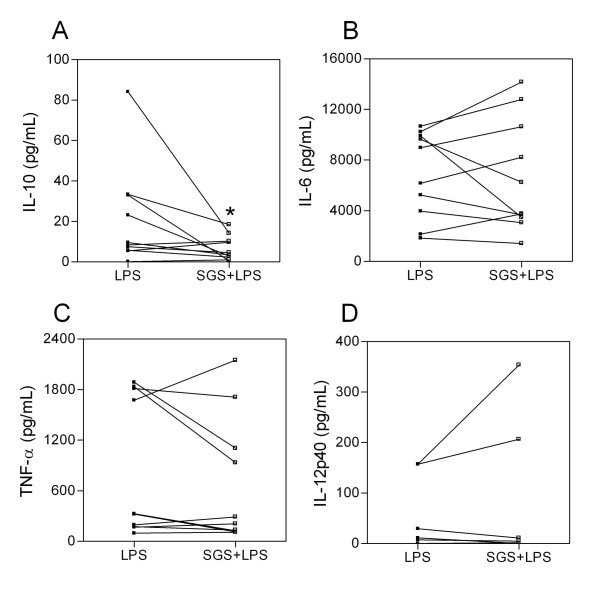
**Effect of *L. intermedia *SGS on LPS-stimulated human monocytes**. Monocytes were pre-treated with SGS overnight and stimulated with LPS for 24 h (TNF-α and IL-6) or 48 h (IL-10 and IL-12p40). The presence of IL-10 (A), IL-6 (B), TNF-α (C) and IL-12p40 (D) was analyzed by ELISA. The data for individual donors are presented. (*p < 0.05). (n = 10)

Within their saliva, sand flies have evolved an array of potent pharmacological components which induce a favorable microenvironment for adequate blood feeding and which may also be important for parasite establishment (rev. in [16]). *L. intermedia *is the sand fly species responsible for the transmission of *Leishmania braziliensis*, the causative agent of cutaneous and mucosal leishmaniasis; thus, we have investigated the effects of *L. intermedia *saliva on the host's immune response in terms of surface molecule expression and cytokine production. Initially, we found that human monocytes stimulated with *L. intermedia *SGS alone did not secrete detectable levels of IL-12p40 or of TNF-α (data not shown). For that reason, we opted to set up an experimental system in which human monocytes were pre-treated with *L. intermedia *SGS and were then stimulated with LPS. In cultures that were pre-treated with SGS, we observed a significant decrease in IL-10 production without significant changes in terms of IL-6, TNF-α or IL-12p40 production (Fig. 1). Modulation of cytokine profiles following exposure to sand fly SGS has already been described; *P. papatasi *saliva was shown to decrease the production of type 1 cytokines and to enhance the production of IL-6 by monocytes [14]. Sand fly saliva from three different species was able to suppress the mitogen-induced proliferative response of murine spleen cells [17]. Last, Costa et al. [15] showed, using the same experimental system employed herein, that *L. longipalpis *SGS inhibits TNF-α and IL-10 production while augmenting IL-6, IL-8, and IL-12p40 production. In that work, it was proposed that components in that sand-fly saliva may promote the development of a cell-mediated immune response through the up-regulation of IL-12 and inhibition of IL-10 production. To that end, development of a delayed-type hypersensitivity (DTH) response was associated with protection against leishmaniasis induced by immunization with SP15, a protein present in *P. papatasi *saliva [18].

**Figure 2:**
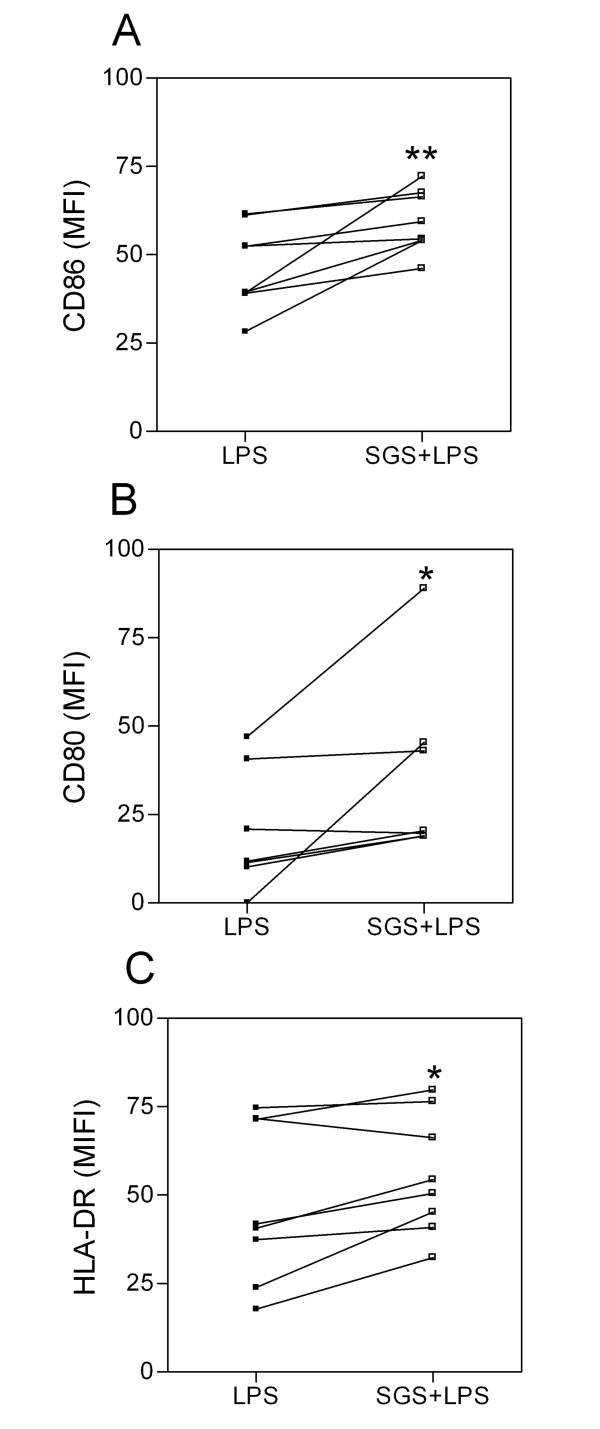
**Effect of *L. intermedia *SGS on co-stimulatory molecule expression on LPS-stimulated human monocytes**. Monocytes were pre-treated with SGS and stimulated with LPS for 48 h. Cells were collected and surface molecule expression was analyzed by flow cytometry. Mean fluorescence intensity of expression is shown for each individual tested (*p < 0.05). (n = 8).

**Figure 3:**
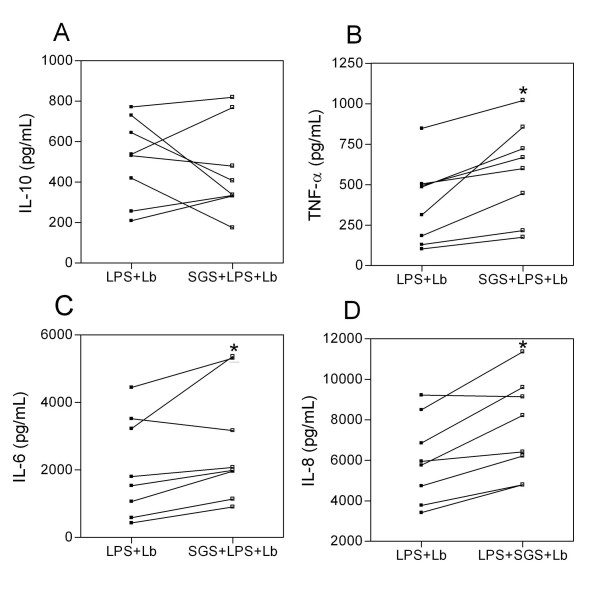
**Effect of *L. intermedia *SGS on cytokine production of LPS-stimulated human monocytes infected with *L. braziliensis***. Monocytes were pre-treated with SGS overnight, stimulated with LPS for 4 h and infected with *L. braziliensis *for 4 hours. The presence of IL-10 (A), TNF-α (B), IL-6 (C) and IL-8 (D) was analyzed by ELISA. The data for individual donors are presented. (*p < 0.05). (n = 8).

**Figure 4:**
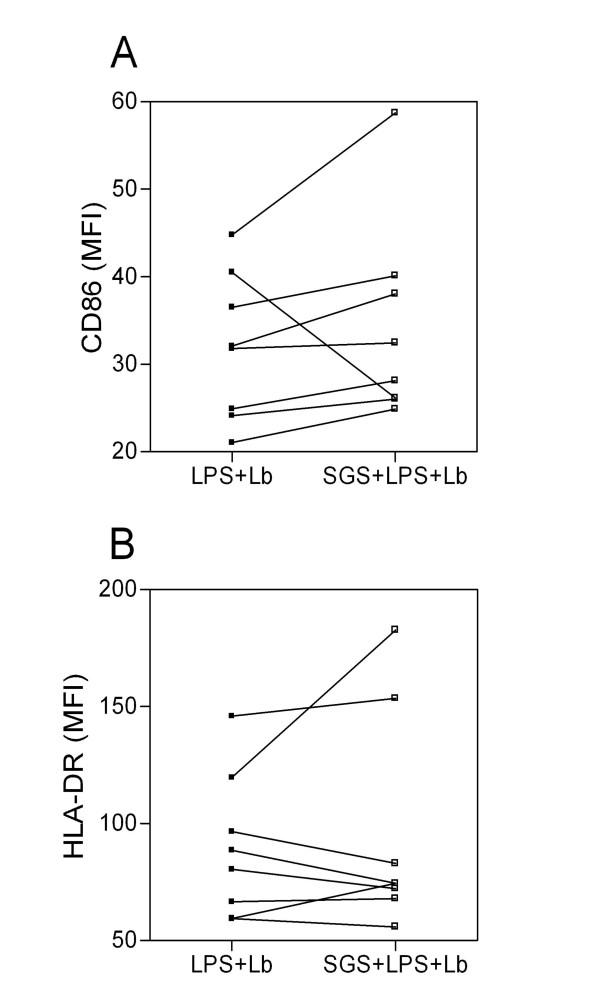
**Effect of *L. intermedia *SGS on co-stimulatory molecule expression of LPS-stimulated human monocytes infected with *L. braziliensis***. Monocytes were pre-treated with SGS overnight, stimulated with LPS for 4 h and infected with *L. braziliensis *for 4 hours. Cells were collected 48 h later and surface molecule expression was analyzed by flow cytometry. Mean fluorescence intensity of expression is shown for each individual tested (*p < 0.05). (n = 8).

**Figure 5:**
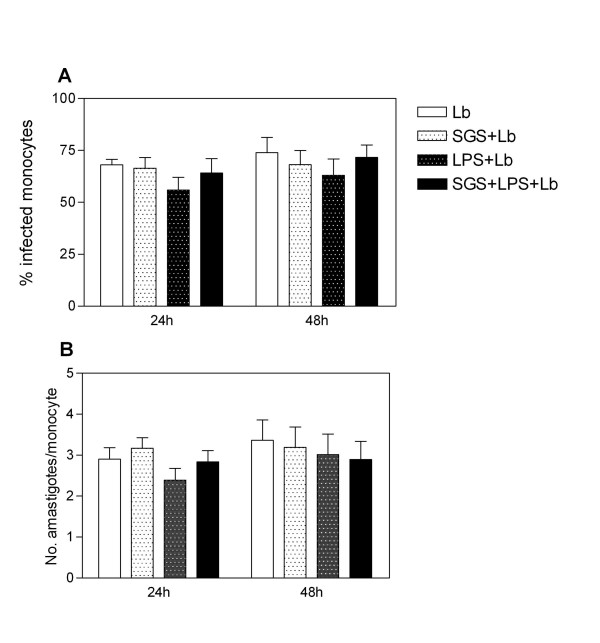
**Effect of *L. intermedia *SGS on parasite load of LPS-stimulated human monocytes infected with *L. braziliensis***. Monocytes were pre-treated with SGS overnight, stimulated with LPS for 4 h and infected with *L. braziliensis *for 4 hours. Glass coverslips were stained with H&E and counted for the number of infected cells (A) and amastigotes per 100 cells (B) by light microscopy. The data are reported as the mean ± the standard error of the mean. (n = 8).

Co-stimulatory molecules play an essential role in the activation and maintenance of T-cell responses. We thus analyzed whether pre-exposure to *L. intermedia *saliva had any effect on CD80, CD86, and HLA-DR expression. Monocytes were pre-treated with SGS and stimulated with LPS, and surface molecule expression was analyzed by flow cytometry. Surprisingly, pre-treatment with SGS was able to significantly increase the mean fluorescence intensity (MFI) of CD80, CD86, and HLA-DR on human monocytes (Fig. 2). Such up-regulation of co-stimulatory molecule expression was not observed with *L. longipalpis *SGS [15], indicating once again that salivary components of these two sand fly species induce different immunomodulatory effects. We can speculate that the increase in co-stimulatory molecule expression observed with *L. intermedia *saliva may be related to the decreased levels of IL-10 (Fig. 1) since it was shown that interleukin-10 differentially regulates B7-1 (CD80) and B7-2 (CD86) expression on human peripheral blood dendritic cells [19]. Class II MHC molecules are required for the activation of CD4 T cells and protection against *L. major *[20]. CD80, CD86, and CTLA-4 also influence the immune response to *Leishmania *[21,22]. The increased expression of co-stimulatory molecules also enhances antigen presentation that, ultimately, may lead to increased T cell activation and IFN-γ production. Indeed, cutaneous leishmaniasis is associated with an intense inflammatory reaction [23,24]. Given these results, we then investigated whether the immune modulation exerted by *L. intermedia *saliva would affect parasite load upon monocyte infection with *L. braziliensis*. To address this hypothesis, human monocytes were pre-treated with *L. intermedia *saliva, stimulated with LPS, and then infected with *L. braziliensis*. Notably, we observed a significant increase in TNF-α, IL-6, and IL-8 production upon parasite infection in cultures pre-treated with SGS, whereas IL-10 levels did not change (Fig. 3). We did not detect significant differences in CD80, HLA-DR (Fig. 4) or in CD86 expression (data not shown). However, up-regulation of the production of TNF-α, IL-6, and IL-8 following pre-treatment with *L. intermedia *SGS and *L. braziliensis *infection did not change the parasite load as measured by the number of infected monocytes (Fig. 5A) or by the number of amastigotes per infected monocyte (Fig. 5B).

Norsworthy et al. [9] also failed to detect significant changes in the infection rate of *L. amazonensis *in murine macrophages that were pre-treated with *L. longipalpis *saliva, despite the significantly higher IL-10 mRNA levels observed. However, addition of Maxadilan, the potent vasodilatory peptide present in *L. longipalpis *saliva, alone was able to up-regulate IL-10, IL-6 and TGF-β and to increase the *L. major *parasite load [25]. We are currently investigating *L. intermedia *and *L. longipalpis *saliva in terms of differences or similarities in immunomodulatory compounds and preliminary results indicate that Maxadilan is not abundant in *L. intermedia saliva *(de Oliveira et al., manuscript in preparation). SDS-PAGE profiles of salivary proteins from both sand fly species showed bands migrating at similar molecular weights, although *L. intermedia *immune sera does react with any protein present in *L. longipalpis *SGS with the exception of the ~45 kDa protein [13], indicating differences in the immunogenicity of salivary molecules.

It is well known that Th1 mediated immunity is important for the control of *Leishmania *infection and that oxidants produced by IFN-γ activated macrophages are the main final effector molecules killing *Leishmania *(rev. in [26]. Although, in this report, we observed an increased production of TNF-α upon pre-treatment of monocytes with *L. intermedia *SGS, this was not associated with decreased *L. braziliensis *infection. This effect may be explained by the parallel up-regulation of both IL-6 and IL-8 production. IL-6 is produced by cells of both the innate and adaptive immune responses, and it was recently shown that IL-6 is able to drive the differentiation of CD4^+ ^Th2 cells by the early induction of IL-4 in CD4^+ ^T cells [27]. IL-8 guides polymorphonuclear cell recruitment to the site of infection, and neutrophils have been shown to act as "Trojan horses" [28] for *Leishmania *establishment. Indeed, development of clinically evident lesions occurs in parallel with the influx of inflammatory cells, including neutrophils, eosinophils and macrophages [29]. Using the air pouch model of inflammation, we observed a significant increase in the recruitment of polymorphonuclear cells upon injection of *L. intermedia *SGS (de Moura et al., manuscript in preparation). In mice exposed to *L. intermedia *SGS, a further inoculation of *L. intermedia *SGS induced an important inflammatory response comprised of numerous polymorphonuclear and few mononuclear cells [13]. We can thus hypothesize that the increase in IL-8 production induced by *L. intermedia *saliva may lead to neutrophil recruitment and, consequently, the successful establishment of infection. Moreover, it will also be interesting to determine whether *L. intermedia *saliva also exerts effects on the effector functions of T-lymphocytes, employing, for example in vitro priming systems [30,14].

## Conclusion

We can conclude that the effects exerted by *L. intermedia *saliva on human monocytes are different from those observed with *L. longipalpis *saliva [15], Maxadilan alone [31,25] and *P. papatasi *saliva [14] and such differences may play an important role regarding the outcome of leishmaniasis. Moreover, since it has been proposed that vaccinating against sand fly salivary components can protect the host from infection (rev. in [32]), our observations also suggest a note of caution regarding the development of vaccines based on sand fly saliva.

## Methods

### Sand Flies and Preparation of SGS

*Lutzomyia intermedia*, Corte de Pedra strain, and *Lutzomyia longipalpis*, Cavunge strain, were reared at Centro de Pesquisas Gonçalo Moniz-FIOCRUZ, as described elsewhere [33]. Adult sand flies were used for dissection of salivary glands 3–5 days after emergence. Salivary glands were stored in groups of 20 pairs in 20 μl NaCl (150 mM) Hepes buffer (10 mM, pH7.4), at -70°C. Immediately before use, salivary glands were disrupted by ultrasonication in 1.5 ml conical tubes. Tubes were centrifuged at 10,000 × g for 2 min and the resultant supernatant (Salivary Gland Sonicate – SGS) was used for the studies. The level of LPS contamination of SGS preparations was determined using a commercially available LAL Chromogenic Kit (QCL-1000, Lonza Bioscience); LPS concentration was <0.1 ng/ml.

### Monocyte Culture

In this work, leukocyte concentrates from healthy adult blood donors were provided by the Blood Bank (HEMOBA) from Salvador, Bahia. Informed verbal consent was obtained from blood donors (six male, four female, age range 30 to 39 years old)

at the time of donation, and all procedures were approved by the local Ethics Committee (CEP/CPqGM-FIOCRUZ) and were conducted following recommendations outlined in the Helsinki Declaration. PBMC were isolated by Ficoll-Hypaque (Sigma) density gradient separation. Cells were then washed, and monocytes were obtained by positive selection with CD14 magnetic beads (Miltenyi Biotech), following the manufacturer's instructions. Briefly, a total of 10^8 ^cells were resuspended in MACS buffer (PBS/0.5% BSA/2 mM EDTA) containing CD14 microbeads. After incubation for 30 min at 4°C, cells were washed and resuspended in MACS buffer and loaded onto MiniMACS MS+ Separation Columns (Miltenyi Biotech). Non-adherent cells were recovered and the total number of CD14-positive monocytes was determined by FACS. The average yield was usually between 92 and 98%. Purified human monocytes were resuspended in complete RPMI (RPMI supplemented with HEPES Buffer 11203, L-glutamine (2 mM), penicillin (100 U/mL), streptomycin (1%) and fetal calf serum (10%), all from Life Technologies) and were pre-treated with *L. intermedia *SGS (equivalent to 2 pairs of salivary gland/ml or 1 ug/ml of protein) for 12 h, followed by stimulation with 20 pg/ml LPS (*E. coli *O111:B14 from Sigma). Cultured cells and culture supernatants were collected 24 h and 48 h later. Co-stimulatory molecule expression was analyzed by flow cytometry and cytokine profiles were determined by ELISA.

### Flow cytometry

Reagents for staining cell surface markers and intracellular cytokines were purchased from BD Biosciences, San Diego, CA. Cells were blocked with anti-Fc receptor antibody (2.4G2) and were stained with anti-human CD80 (L307.4), CD86 (2331) and HLA-DR, DP and DQ (G46-6) conjugated to PE. Isotype controls were used as appropriate. For each sample, 20,000 events were analyzed using CELLQuest™ software and a FACSort^® ^flow cytometer (Becton Dickinson Immunocytometry).

### Cytokine assays

Concentrations of TNF-α, IL-6, IL-8, IL-10 and IL-12p40 in culture supernatants were determined by ELISA using commercial kits (BD Pharmingen), following the manufacturer's instructions.

### Parasite culture

*L. braziliensis *strain MHOM/BR/01/BA788 was isolated from a patient with cutaneous leishmaniasis from the state of Bahia (northeastern Brazil) after brief (2–4) passages in culture medium. This isolate was identified as *L. braziliensis *by using PCR [34] and monoclonal antibodies [35]. Promastigotes were grown in Schneider medium (Sigma) supplemented with 100 U/ml of penicillin, 100 ug/ml of streptomycin and 10% heat-inactivated fetal calf serum (all from Life Technologies). Monocytes were pre-treated with SGS as above for twelve hours, stimulated with LPS for four hours and were infected with *L. braziliensis *parasites (5 parasites to 1 monocyte) for four hours. Infected monocytes were washed for removal of parasites and cultivated for 24 and 48 h. Co-stimulatory molecule expression was analyzed by flow cytometry and cytokine profiles were determined by ELISA.

### Statistical analysis

Statistical analyses comparing cytokine responses and surface molecule expression were performed using Prism (GraphPad Software). Data were compared using the Wilcoxon signed-rank test. In all cases, results were considered significantly different when the *P*-value was < 0.05.

## Authors' contributions

MJM and JC performed experiments and analyzed data. JC carried out flow cytometry analysis. JCM contributed materials to the study. AB and MBN participated in study design and helped to draft the manuscript. CB participated in study design and data analysis. CIO conceived of the study, and participated in its design and coordination and drafted the manuscript. All authors read and approved the final manuscript.
